# Two-Dimensional Blue Native/SDS Polyacrylamide Gel Electrophoresis for Analysis of Brazilian *Bothrops* Snake Venoms

**DOI:** 10.3390/toxins14100661

**Published:** 2022-09-23

**Authors:** Natacha Ferreira de Oliveira, Ana Teresa Azevedo Sachetto, Marcelo Larami Santoro

**Affiliations:** 1 Laboratório de Fisiopatologia, Instituto Butantan, São Paulo 05503-900, SP, Brazil; 2 Escola Superior do Instituto Butantan (ESIB), Instituto Butantan, São Paulo 05503-900, SP, Brazil; 3 Programa de Pós-Graduação em Ciências Médicas, Faculdade de Medicina, Universidade de São Paulo, São Paulo 01246-000, SP, Brazil

**Keywords:** zymography, collagenolytic activity, amidolytic activity, protein chains, protein subunits, mass spectrometry, Coomassie brilliant blue G-250, botrocetin

## Abstract

Viperidae snakes are the most important agents of snakebites in Brazil. The protein composition of snake venoms has been frequently analyzed by means of electrophoretic techniques, but the interaction of proteins in venoms has barely been addressed. An electrophoretic technique that has gained prominence to study this type of interaction is blue native polyacrylamide gel electrophoresis (BN-PAGE), which allows for the high-resolution separation of proteins in their native form. These protein complexes can be further discriminated by a second-dimension gel electrophoresis (SDS-PAGE) from lanes cut from BN-PAGE. Once there is no study on the use of bidimensional BN/SDS-PAGE with snake venoms, this study initially standardized the BN/SDS-PAGE technique in order to evaluate protein interactions in *Bothrops atrox*, *Bothrops erythromelas*, and *Bothrops jararaca* snake venoms. Results of BN/SDS-PAGE showed that native protein complexes were present, and that snake venom metalloproteinases and venom serine proteinases maintained their enzymatic activity after BN/SDS-PAGE. C-type lectin-like proteins were identified by Western blotting. Therefore, bidimensional BN/SDS-PAGE proved to be an easy, practical, and efficient method for separating functional venom proteins according to their assemblage in complexes, as well as to analyze their biological activities in further details.

## 1. Introduction

Snakes of the Viperidae family are the main cause of snakebite accidents in Brazil [1]. *Bothrops* is the main representative genus of this family in Brazil. In particular, three species account for most accidents: *Bothrops atrox*, which is found in the northern region of Brazil, and is also present in Bolivia, Ecuador, Peru, Venezuela, Colombia, Guyana, French Guiana, and Suriname; *Bothrops jararaca*, a species that inhabits the southeastern region of Brazil, Argentina, and Paraguay; and *Bothrops erythromelas*, a species from the northeastern region of Brazil [2].

Snake venoms are composed of a wide variety of bioactive substances—e.g., carbohydrates, inorganic elements, peptides, carboxylic acids, lipids, biogenic amines, purine nucleosides, and others—but 60–90% of them are proteins [3,4,5,6]. Some of these proteins have been described to form covalent and/or non-covalent complexes with other homologous and/or heterologous proteins [7,8,9], which possibly enhance the pathophysiological action of venoms to paralyze and kill preys. For example, in the rattlesnake *Crotalus durissus terrificus* venom, crotoxin (basic subunit) and crotapotin (acid subunit) form a heterodimeric complex, which dissociates after the complex reaches its target at the pre-synaptic membrane or the sarcolemma [10].

In most *Bothrops* venoms, snake venom metalloproteinases (SVMPs) and snake venom serine proteinases (SVSPs) are the preponderant enzymes present therein [11]. *Bothrops* SVMPs are abundant enzymes, accounting for 42–72% of venom proteins in *B. atrox* [12,13], 10–37% in *B. jararaca* [14,15], and 39–49% in *B. erythromelas* [16]. They have been directly linked to the main effects observed during envenomation, such as blood incoagulability, hemorrhage, inflammation, and myonecrosis. These enzymes are subdivided into groups according to their molecular mass and domain structure: P-I, P-II, and P-III [17,18]. SVSPs account for 4–11% of the venoms of *B. atrox* [12,13], 4–21% of *B. erythromelas* [16], and 13–29% of *B. jararaca* [14,15]. SVSPs have molecular masses of 26–67 kDa, and when isolated they show no toxic effects, but their biological interaction with other venom components causes hemostatic imbalance by interfering in the coagulation cascade, fibrinolysis, and platelet aggregation. For example, the SVSPs ancrod (from *Calloselasma rhodostoma*) and batroxobin (from *Bothrops moojeni*) have thrombin-like activity, and they are used clinically as anti-thrombotic drugs, with no toxicity [19,20]. Phospholipases A_2_ (PLA_2′_s) are low molecular mass enzymes (13–16 kDa) that hydrolyze phospholipids. In snakebites inflicted by *Bothrops* snakes, PLA_2_′s mainly cause inflammation, local pain, edema, and anticoagulant effects [21]. These proteins are not found in large amounts in the venoms of *B. atrox* (4–24% [12,13]), *B. jararaca* (3–20% [14,15]), and *B. erythromelas* (10–15%) [16]. C-Type lectin-like proteins (CTLPs) are a family of non-enzymatic proteins present in *Bothrops* venoms [15,16,22], which can profoundly alter platelet function, blood coagulation, and immune cells. Their basic structures consist of heterodimers, formed from α and β subunits or chains, whose molecular masses are around 14–16 kDa and 13–15 kDa, respectively, covalently linked by disulfide bonds [23]. They are also present as chains of some P-IIId SVMPs—e.g., in the factor X activators from venoms of *Vipera lebetina* [9] and *Daboia russelii* [24], and prothrombin activators from venoms of *Echis multisquamatus* [25,26,27] and *Echis carinatus* [28].

To evaluate the structures of protein complexes, various analytical techniques have been used, including electrophoretic ones [29,30]. Electrophoresis uses an electric field generated by a power supply, so that the electric current between the negative pole (cathode) and the positive one (anode) promotes protein migration according to their molecular masses, electric charges, and/or shapes. Thus, those proteins with lower molecular masses move more easily, whereas those with higher molecular masses migrate less.

**Figure 1:**
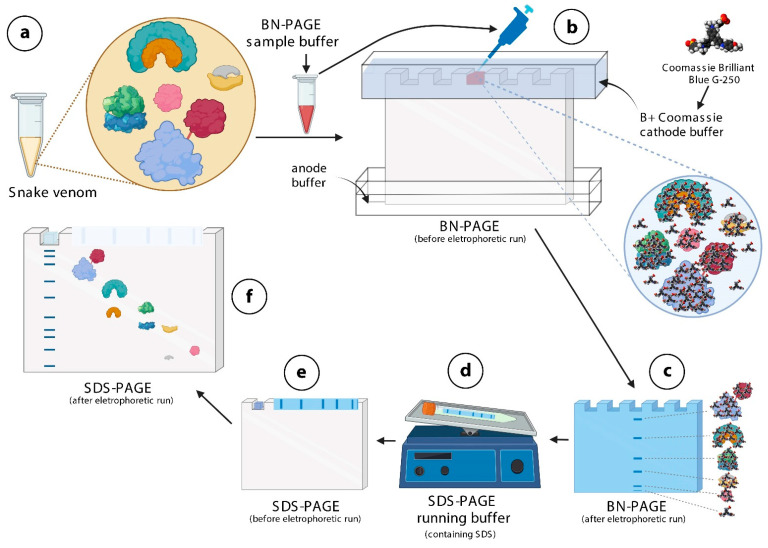
Schematic representation of the mechanistic principle of BN/SDS-PAGE for separation of protein complexes in snake venoms. (**a**)—Snake venoms contain protein complexes. In this example, four different protein complexes and one monomeric protein (pink in color) are shown. The red line between the baby blue, and the red protein indicates a disulfide bond. The snake venom is diluted in BN-PAGE sample buffer containing Ponceau S. (**b**)—This mixture is loaded into the wells of sample application gel, previously filled with B+Coomassie buffer. The Coomassie brilliant blue G-250 dye contained in this buffer binds onto the surfaces of protein complex, not allowing them to dissociate. (**c**)—Protein complexes (blue lines) migrate through the running gel, according to their molecular masses. (**d**)—Lanes are cut and incubated in SDS-PAGE running buffer, so that SDS binds to proteins, conferring tonto hem very similar charge-to-mass ratios. (**e**)—The lane and the filter paper containing the molecular mass markers are then inserted over the running gel, fixed with agarose, and electrophoresed. (**f**)—Except for protein complexes linked by disulfide bonds, the others disassemble and migrate vertically through the running gel, according to their molecular masses. The figure was partially created with biorender.com.

The two-dimensional electrophoresis commonly used today was developed in the 1970s [31], in which proteins are separated first based on their isoelectric points (first dimension), and subsequently they undergo separation based on their molecular masses (second dimension). Although it is very efficient, this technique is very labor-intensive and expensive (it requires special equipment, isoelectric focusing (IEF) strips, and high-cost consumables, such as pharmalytes, iodoacetamide, CHAPS, etc.), and alternative techniques have been used to replace it in several laboratories. In fact, blue native polyacrylamide gel electrophoresis (BN-PAGE) has gained prominence in recent years. It is an electrophoretic technique that allows separation of protein complexes in their native form at high resolution, by using Coomassie brilliant blue G-250, a dye that causes a charge shift on proteins, so that they migrate to the anode. In fact, the dye binds to hydrophobic and arginine acid residues on the surface of proteins, allowing them to move more easily through the running gel [32,33]. Thus, proteins are not separated according to the charge/mass ratio in BN-PAGE, but according to their size. BN-PAGE does not alter proteins, but maintains them in similar native states to those observed in vivo [34] (Figure 1). BN-PAGE has been widely used to isolate and determine the existence of protein complexes from various organisms, as well as to determine native protein masses and oligomeric states [34,35]. To separate protein complex subunits according to their molecular masses, BN-PAGE can be associated with a second-dimension electrophoresis, e.g., sodium dodecyl sulfate (SDS-PAGE) electrophoresis (BN/SDS-PAGE) [32,34,35,36].

As far as we know, there are no articles available in the literature that used BN/SDS-PAGE to evaluate protein complexes in snake venoms. Using electrophoresis techniques to understand the protein composition of snake venoms is a common practice in the laboratory, and when subsequently associated with zymographic or blotting analyses, they may provide insight into the biological properties of snake venom and correlate them with their pathophysiologic actions. BN/SDS-PAGE provides more information than unidimensional SDS-PAGE or bidimensional IEF/SDS-PAGE: it allows for verification of the formation of protein complexes in native states. In fact, little is known about the interactions between diverse proteins in snake venoms, and whether these interactions can somehow influence the mechanism of action of these proteins.

Thus, this work first aimed to standardize BN/SDS-PAGE to evaluate the venoms from three of the most important agents of snakebites in Brazil: *B. atrox*, *B. erythromelas*, and *B. jararaca*. In a second step, it was evaluated whether these separated proteins still showed proteolytic and esterase activity when analyzed by zymography. Our results showed that BN/SDS-PAGE, zymography, and Western blotting are all simple and non-expensive techniques that provide useful information about the composition and formation of protein complexes in *Bothrops* snake venoms.

## 2. Results and Discussion

BN/SDS-PAGE was initially described in 1991 for the separation of membrane protein complexes [37], and thereafter it has been extensively applied in other studies [32], allowing for the visualization of protein complexes from different organisms. As shown underneath, BN/SDS-PAGE proved to be a very effective and rapid technique, allowing visualization of protein complexes in their native form, and preservation of the biological activities of SVMPs and SVSPs. BN/SDS-PAGE combined with Western blotting also provided visualization of CTLPs, and assessment of the immunoreactivity of commercial antivenins.

**Figure 2:**
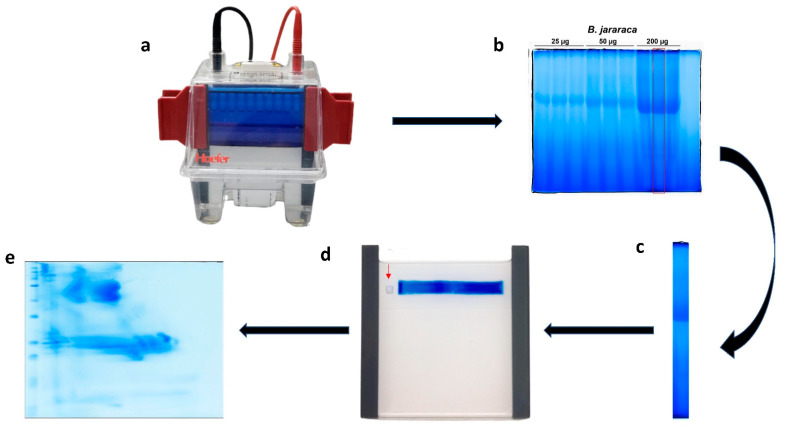
Schematic representation of BN/SDS-PAGE. (**a**) The first-dimension run was halted when the blue dye front reached 1/3 of the running length. Following that, the cathode B+Coomassie buffer was removed, the cathode B/10 buffer was poured. (**b**) After the end of the first-dimension electrophoresis, lanes were individually cut with a spatula. (**c**) After incubation with SDS-containing buffer and rinsing, (**d**) the lane was inserted between plates for the second-dimension run; the molecular mass standard (square filter paper, red arrow) was standardized to be maintained on the left side of the first-dimension lane. (**e**) After the second-dimension run (SDS-PAGE), the gels can be analyzed by several techniques. In this image, *B. jararaca* venom was stained with blue silver [38].

Schemes and photos of how to perform BN/SDS-PAGE on snake venom samples are depicted in Figure 1 and Figure 2. Two different cathode buffers are used in the first-dimension separation (Figure 2a,b). When the first electrophoresis run finishes, the lanes can be immediately visualized, and then they are cut and frozen (Figure 2b,c). After SDS-PAGE gel has been polymerized, the lane is thawed, incubated in sample buffer containing SDS (Figure 1d), and electrophoresed (Figure 1e and Figure 2d). Thereafter, the gel can be stained for proteins (Figure 1f and Figure 2e), and analyzed by zymography, mass spectrometry, or Western blotting.

### 2.1. First-Dimension Electrophoresis (BN-PAGE)

Six final amounts of *B. jararaca* venom were initially tested: 10, 25, 50, 100, 150, and 200 μg. The best results were obtained using 200 µg of the venom sample. This finding shows that higher amounts of protein can be loaded into BN/SDS-PAGE than into IEF/SDS-PAGE. In fact, for the same electrophoresis cube used herein for BN/SDS-PAGE, the maximum amount of *B. jararaca* venom that could be loaded into IEF strips was 80 µg (for a 7 cm IEF strip) [4]. An advantage of the higher amounts of loaded proteins in BN/SDS-PAGE is that it allows the detection of low abundant proteins—e.g., glutaminyl-peptide cyclotransferase, carboxypeptidase, PLA_2_ inhibitor, and beta-defensins—by mass spectrometry (see below).

The ideal polyacrylamide gel percentage was determined to be 12% in the first-dimension run (Figure 2b), as at lower percentages of polyacrylamide (5 or 10%) the proteins ran through the gel with greater mobility, and at higher acrylamide percentages (15%) they accumulated at the top of the running gel (data not shown). We also tested polyacrylamide gradient gels (4–13%) for the first-dimension gels, but we could not observe any evident differences in regard to protein separation to 12% polyacrylamide gels (data not shown). Thus, searching for an easy and rapid technique, we chose to use non-gradient gels, as they can be easily performed in laboratories, even if there are limitations in resources. The venoms of *B. atrox*, *B. jararaca*, and *B. erythromelas* showed the same pattern of protein migration shown in Figure 2c, i.e., one band was more intensely stained, localized at relative migration distances (R_f_) of 0.45–0.46.

In addition to the BN/SDS-PAGE performed with the three venom samples used in this study, venoms from other snake species—*C. durissus terrificus*, *Daboia russelii*, *Bothrops jararacussu* and *Echis carinatus*—were also tested. However, the results were not satisfactory because proteins from these venoms accumulated on the edge of the running gel during the first-dimension electrophoresis (data not shown). In these venoms, the Coomassie brilliant blue G-250 dye seemed to bind to proteins in a diverse way, aggregating them and not allowing their entrance into the polyacrylamide gel. Other proportions of the dye were also tested, but no positive results were obtained with these venoms (data not shown). Further studies are required to understand this phenomenon.

### 2.2. Profiles of Protein Spots in Venoms Analyzed by BN/SDS-PAGE

**Figure 3:**
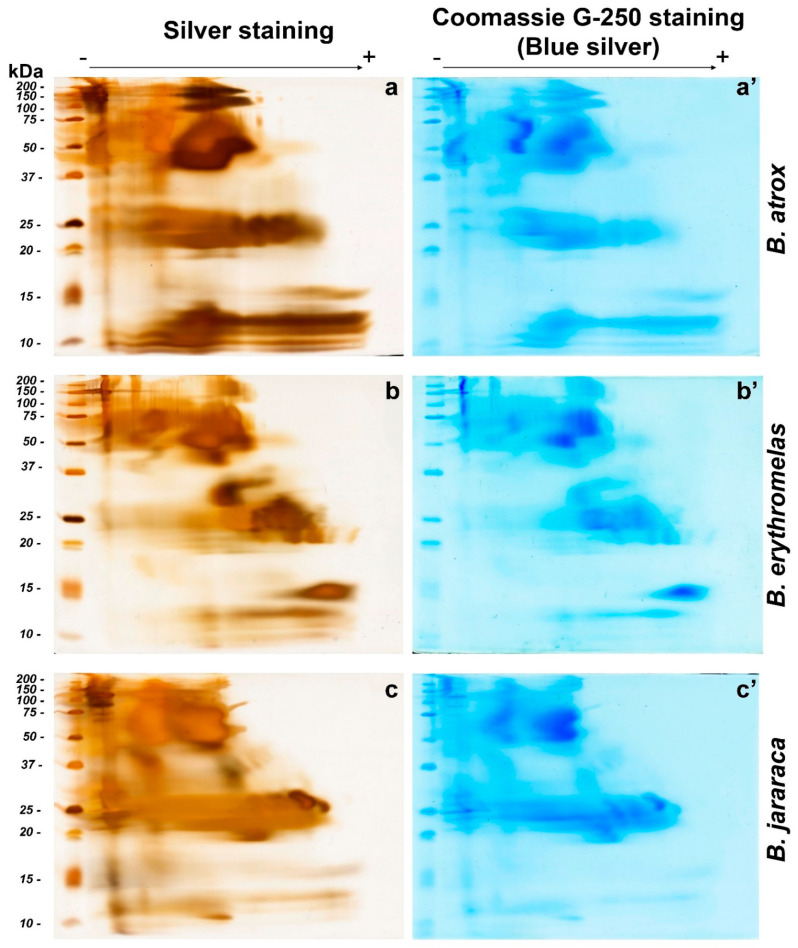
Comparison of BN/SDS-PAGE profiles of *B. atrox* (**a**,**a’**), *B. erythromelas* (**b**,**b’**), and *B. jararaca* (**c**,**c’**) venoms. Gels were run under identical conditions, and stained first with silver nitrate (left column: (**a**–**c**)) [40], scanned, and destained [41]. Thereafter, the same gels were stained with blue silver (right column: (**a’**–**c’**)) [38] and scanned. Observe that spots are less evident in blue silver-stained gels than in those stained with silver nitrate.

After performing BN/SDS-PAGE, similarities were noticed between venoms. It was noticed that the protein migration profiles were very replicable. As discussed above, higher amounts of protein could be loaded into BN/SDS-PAGE. A disadvantage of this is the coalescence between neighbor spots, giving the impression of protein smears in BN/SDS-PAGE, In fact, proteins migrated to the positive pole, demonstrating that they had negative charges, as expected by the binding of Coomassie brilliant blue G-250 to proteins [32]. However, most proteins had only migrated up to the middle of the running gel (from 200 to 20 kDa) as the dye marker reached the end of the run (Figure 3a–c). Thus, after the second-dimension run, proteins are concentrated into basically three blocks: one with proteins from 200 to 37 kDa; another with proteins from 30 to 19 kDa; and the third with proteins from 15 to <10 kDa. This pattern was observed for gels stained with either silver nitrate (Figure 3a–c) or blue silver (Figure 3a’–c’). However, small differences were observed between silver nitrate and blue silver stains. In blue silver-stained gels, not all spots were as evident as in those stained by silver nitrate, inasmuch as silver nitrate is more sensitive to lower amounts of proteins than blue silver, and their staining principles are diverse [39].

In *B. atrox* venom (Figure 3a,a’), the two most prominent spots were observed between 37 and 75 kDa, in the middle of the first-dimension run. Immediately above these bands, two smaller spots were present between 100 and 150 kDa. The latter spots, compared to the other two venoms, were more intensely stained. Spots with molecular masses between 20 and 25 kDa were also intensely stained, but they were not clearly separated from each other. Elongated protein spots, with molecular masses ranging from 10 to 15 kDa, were also observed in *B. atrox* venom, and were more intensely stained compared to *B. jararaca* and *B. erythromelas* venoms. These elongated spots were not so clearly evident by blue silver staining (Figure 3a’) compared to silver staining (Figure 3a).

In the silver-nitrate-stained gel from *B. erythromelas* venom (Figure 3b), intensely stained spots were evident between 37 and 75 kDa, and were more clearly separated compared to the other two venoms. A spot between 25 and 37 kDa was intensely stained in the venom of *B. erythromelas*, more faintly stained in *B. jararaca*, and completely absent in *B. atrox*. Between 20 and 25 kDa, various spots could be distinguished, and they were well separated from each other. At 15 kDa, an intensely stained positively charged spot was visualized, and it was faintly stained in the other venoms.

*Bothrops jararaca* venom had intensely stained spots between 100 and 150 kDa (Figure 3c,c’). The spots with molecular masses between 37 and 75 kDa are more abundant and larger in size compared to the other two venoms. Between 20 and 30 kDa, the spots were elongated, and more intensely stained than in the venoms of *B. atrox* and *B. erythromelas*. Two elliptical 25–30 kDa spots, localized closer to the positive pole, were more clearly observed in *B. jararaca* venom than in the venoms of *B. atrox* and *B. erythromelas*. Below 15 kDa, spots were elongated and not clearly evident as in the venoms of *B. atrox* and *B. erythromelas*.

### 2.3. Mass Spectrometry

**Figure 4:**
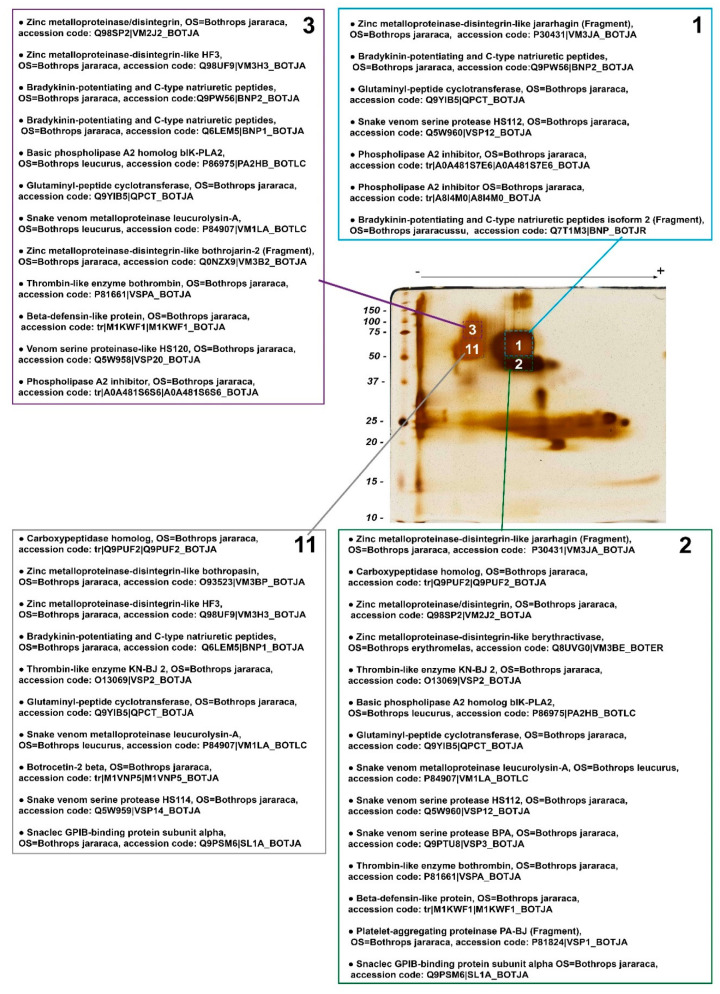
Mass spectrometry identification of snake venom proteins present in the 4 most evident spots (#1, 2, 3, and 11) selected from a BN/SDS-PAGE gel of *B. jararaca* venom-stained silver nitrate. Identified proteins were classified in decreasing order in each list, according to—10 logP score, i.e., the scoring significance of a peptide-precursor spectrum. Proteins that were identified, but are not constitutively found in snake venoms were excluded. The complete list of identified proteins can be found in the Supplementary Table S1. OS: Organism name.

Identification of proteins present in four selected spots of *B. jararaca* snake venom by LC-MS/MS showed that each spot was comprised by a mixture of proteins, each containing at least one SVMP, one SVSP, and glutaminyl-peptide cyclotransferase (Figure 4). According to the mechanistic principle of BN/SDS-PAGE (Figure 1), this finding demonstrates that these proteins are likely to form complexes in native states. Although one spot might contain proteins that form complexes with others therein, they might also form complexes with proteins that are immediately above or below them, in vertical lines (Figure 1). Herein, we just addressed the question of whether there are putative conditions to form protein complexes in snake venoms, and according to the results obtained by BN/SDS-PAGE and MS, the existence of such protein complexes is likely. These findings are corroborated by zymography images (see below), since these four spots showed amidolytic and collagenolytic activity. Interestingly, glutaminyl-peptide cyclotransferase (43 kDa), an enzyme that catalyzes N-terminal pyroglutamate (pE) formation on proteins or peptides, was present in the four spots, showing its interaction and relevance within the venom. pE formation is required for structural stability, resistance to aminopeptidase degradation, and interaction between proteins [42,43]. 

PLA_2_ inhibitors were noticed in spots 1 and 3, whereas basic PLA_2_ homologs were present in spots 2 and 3, and thus putative complexes between those two families of proteins might occur within spot 3, and between spots 1 and 2, which are vertically associated.

Two CTLPs—the α chain of GPIb-binding protein [44] and the β-chain of botrocetin 2 [45]—were found in spots 2 and 11, whose molecular masses are above those of single heterodimers, suggesting that CTLPs may be moieties of P-IIId SVMPs or form oligomers. Although the formation of more complex structures with higher molecular masses for botrocetin or GPIb-binding protein has not been demonstrated in the literature, other native CTLPs—e.g., rhodocetin [46] and convulxin [47]—have been shown to form heterodimers, heterotrimers, and heterotetramers. Similarly, the precursors of bradykinin potentiating and C-type natriuretic peptides [48], carboxypeptidase, and β-defensin-like protein [49] were identified in spots whose molecular masses are much higher than those expected theoretically. These findings indicate that these proteins may form complexes with other proteins.

### 2.4. Zymography

#### 2.4.1. Amidolytic Activity

**Figure 5:**
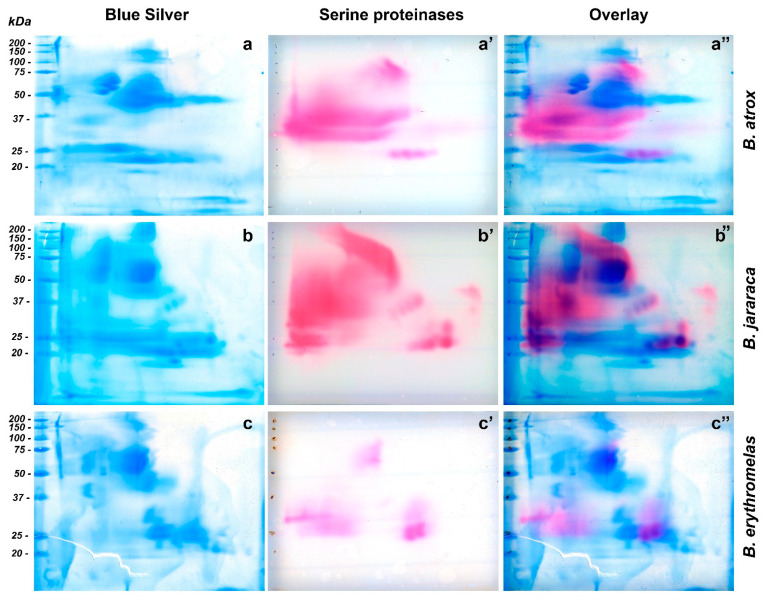
Comparison of two-dimensional gel electrophoresis profiles of *B. atrox* (**a**,**a’,a’’**)*,* *B. jararaca* (**b**,**b’,b’’**), *B. erythromelas* (**c**,**c’,c’’**). Gels were run under identical conditions. To provide the location of SVSP amidolytic activity (pink, (**a’**,**b’**,**c’**)) in blue silver-stained gels (blue, (**a**–**c**)), images of scanned membranes and gels were overlaid on Photoshop CS6 (pink/blue, (**a’’**,**b’’**,**c’’**)). Most of the SVSP staining is not evident on gels stained for proteins with blue silver.

As far as we know, the methodology used herein to observe SVSP activity [50] in snake venoms has not been employed yet. By means of that, we could observe a diversity in spot arrangements and enzyme activity among venoms, which demonstrates that the distribution of SVSPs, even within the same genus, is diverse. In fact, the pink areas were larger and more intense in venoms from *B. atrox* and *B. jararaca* than from *B. erythromelas*, corroborating previous reports that *B. erythromelas* has lower SVSP activity [51,52], even though the three venoms show a similar content of SVSP in their venoms [12,13,14,15,16]. The low SVSP activity is likely caused by mutations or deletion of SVSP genes in *B. erythromelas* [53]. Interestingly, by overlaying images from SVSP zymography and blue silver-stained gels, the position of the SVSP bands could be confirmed, and it became evident that SVSPs were faintly stained by silver nitrate and blue silver (Figure 5).

*Bothrops atrox* venom showed great SVSP activity (Figure 5a’), and three major regions were noticed in nitrocellulose membranes. The first one showed a large staining area, between 30 and 50 kDa, with a predominance of pink staining towards the left side (cathode) of the nitrocellulose membrane. Between 20 and 25 kDa (from the central to the anode region), four similar well-defined circular spots had intermediate pink staining. The third region was present in the central region of the membrane, whose molecular masses were around 75–150 kDa (Figure 5a). By overlaying images (Figure 5a”), the only bands that were noticed to superpose were those between 75–150 kDa, and the four bands between 20–25 kDa. The most intense SVSP staining (30–50 kDa) was in a region where proteins were minimally stained by silver or blue silver staining (Figure 5a). SVSPs have been shown to be highly glycosylated [54], and that is likely the reason why they are barely visible by protein stains.

In the membrane of *B. jararaca* venom (Figure 5b’), SVSP staining was intense and appeared well distributed over the range of molecular masses of proteins, but spots tended to concentrate from the center of the membrane towards the left side (cathode). At 50–150 kDa, a very characteristic left-to-right diagonal band was observed. A major, intensely stained 30–50 kDa spot developed from the cathode border to the central region. At around 23–24 kDa, three intensely stained, horizontally distributed circular spots were visualized from the center to the right side (anode) of the membranes; immediately above the rightmost spot, there was one 29-kDa spot. The four spots (20–25 kDa) that were observed in *B. atrox* venom also showed the same migration as that of *B. jararaca* venom, but their molecular masses were between 33 and 39 kDa. When images were overlaid (Figure 5b’’), the aforementioned left-to-right diagonal spot (50–150 kDa) in SVSP zymography was not identified by blue silver staining, while the largest spot stained by blue silver (50–75 kDa) (Figure 5b) did not show intense reaction to SVSP activity. The four intensely stained spots (three 23–24 kDa spots, and one 29 kDa spot) were perfectly aligned with proteins stained by blue silver staining, as well as the four diagonal SVSP-stained spots, whose molecular masses were around 33–39 kDa.

In *B. erythromelas* venom (Figure 5c’), three major areas of staining were observed, although they were less intensely stained compared to *B. jararaca* and *B. atrox* venoms. The first area contained central spots around 50–75 kDa; in the second one, spots, whose molecular masses were around 25–32 kDa, were distributed around the left border (cathode); in the third area, proteins migrated more to the anode, and two major spots (around 25 kDa) and one large 35 kDa spot were observed. Proteins in the third area were also observed in *B. jararaca* and *B. atrox* venoms. By overlaying images (Figure 5c’’), most proteins of zymography-stained spots (Figure 5c’) did not superpose with proteins stained by blue silver (Figure 5c), except for the spots (around 25 kDa) in the third area that showed correspondent spots in blue silver.

Among the tests performed, we also tested the chromogenic substrate Nα-Benzoyl-DL-arginine 4-nitroanilide hydrochloride (BAPNA), commonly used to verify the SVSP activity [4]. However, BAPNA did not provide good staining, as the color development was very faint, even after long incubation periods.

#### 2.4.2. Gelatinolytic Activity

SVMPs are the most abundant enzymes present in *Bothrops* venoms. However, when venom proteins were separated by BN/SDS-PAGE and subsequently gelatinolytic zymography was carried out, few lytic areas were observed compared to SVSP zymography.

**Figure 6:**
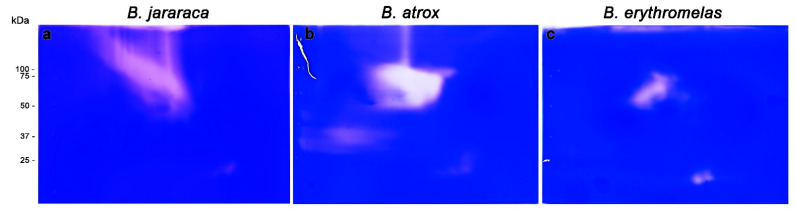
Gelatinolytic activity of *B. jararaca* (**a**), *B. atrox* (**b**), and *B. erythromelas* (**c**). Venoms were submitted to BN/SDS-PAGE under the same conditions and stained with Coomassie brilliant blue R-250. The clear areas correspond to the gelatinolytic activity in the gels.

In regard to gelatinolytic areas observed in *B. jararaca* venom (Figure 6a), one large, left-to-right diagonal area (50–150 kDa), and one smaller opaque area (25 kDa), close to the anode, were visualized; they were similar in shape and localization to the spots developed in SVSP zymography (Figure 5b’), exactly superposing when both images were overlaid (data not shown). 

In *B. atrox* venom (Figure 6b), three lytic areas were observed: a major central lytic area, between 50–75 kDa; a minor lytic area localized at 25 kDa, similar to that observed in *B. jararaca* venom; and a faint horizontal lytic area (close to the cathode) around 37 kDa. The major gelatinolytic area (50–75 kDa) could be identified in both silver nitrate and blue silver stainings (Figure 3a,a’), but differently from *B. jararaca* venom, the superposition of images of SVSP zymography and gelatinolytic activity did not show high comparability (data not shown).

Unlike the other two venoms, lytic areas were less expressive in *B. erythromelas* venom (Figure 4c). At 50–75 kDa, there was a central area of lysis, but the two 25 kDa spots, which were less evident in *B. jararaca* and *B. atrox* venoms, were more intense in *B. erythromelas* venom.

Altogether, gelatinolytic zymography showed that some spots appeared in positions similar to those found in SVSP zymography, as in *B. jararaca* venom, demonstrating that it is likely that there are protein complexes between these two families of enzymes in *Bothrops* venoms. Such an assumption is demonstrated by the coexistence of SVMPs and SVSPs in the four spots analyzed by MS. Although it has been known since the 1950s that most gelatinolytic activity of *Bothrops* venoms can be ascribed to SVMPs [4,55], at least four SVSPs isolated from *Bothrops* venoms, including one from *B. atrox* and one from *B. jararaca* [54,56,57,58], display gelatinolytic activity. Thus, the results of zymographic analyses are not conclusive of the coexistence of SVMPs and SVSPs in *B. jararaca*. 

It is worth mentioning that various methodological approaches for visualization of PLA_2_ activity were tested, e.g., direct incubation of BN/SDS-PAGE gels or blotted membranes with soybean lecithin [3] or synthetic substrates [59], or by placing gels directly over erythrocyte-yolk agarose [60]. However, these experiments did not evidence any enzymatic reaction. When methods to evaluate PLA_2_ activity after PAGE electrophoresis were searched in the literature, we found none, indicating that PLA_2_‘s lose enzymatic activity after SDS-PAGE.

### 2.5. Western Blotting

**Figure 7:**
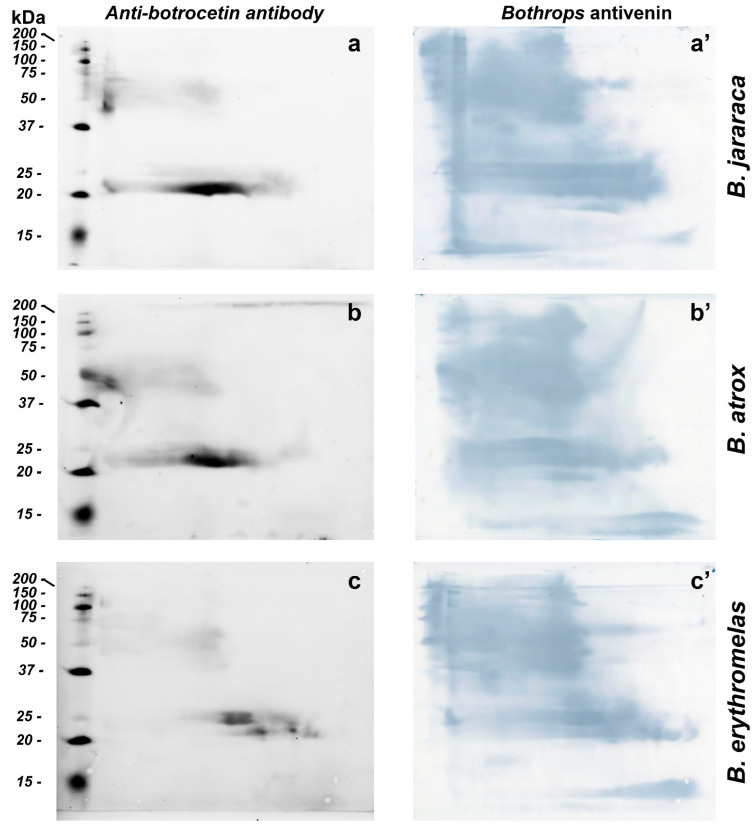
Western blotting of *B. jararaca* (**a**,**a’**), *B. atrox* (**b**,**b’**) and *B. erythromelas* (**c**,**c’**). The venom proteins were subjected to BN/SDS-PAGE. In the left column (**a**–**c**), nitrocellulose membranes were incubated with the anti-botrocetin polyclonal antibody, the anti-rabbit IgG conjugated with AlexaFluor 647, and then analyzed in a fluorescence detecting system. Thereafter, each membrane was incubated with *Bothrops* antivenin and developed with DAB (right column, (**a’**–**c’**)).

BN/SDS-PAGE and Western blotting were used for identification and evaluation of venom proteins without proteolytic activity (e.g., CTLP). For the identification of CTLP, we used polyclonal antibodies raised in rabbits against botrocetin, a CTLP from *B. jararaca* venom [61]. Anti-botrocetin polyclonal antibodies detected similar intense spots in *B. atrox* and *B. jararaca* venoms, between 20 and 25 kDa at the center of the membrane, but the elongated spot was more prominent in *B. jararaca* venom (Figure 7a–c). Fainter spots, between 50 and 75 kDa, were also present in all three venoms tested, and confirmed the presence of botrocetin by MS in spots 2 and 11 (Figure 4). As discussed above, botrocetin chains may be moieties of P-IIId SVMPs or form oligomers. In *B. erythromelas*, this antibody recognized fainter spots between 20 and 25 kDa, localized closer to the cathode.

*Bothrops* antivenin is potent enough to neutralize different classes of proteins found in *Bothrops* venoms [14], and could recognize with high sensitivity different classes of proteins from the three venoms tested (Figure 7a’–c’). After fluorescence scanning of CTLPs using anti-botrocetin antibodies, the nitrocellulose membranes were incubated with *Bothrops* antivenin, and the reaction was developed using DAB. The sensitivity of *Bothrops* antivenin to detect proteins in nitrocellulose membranes was higher than that of either silver nitrate or blue silver staining in gels. Interestingly, proteins that were hardly detected by silver nitrate or blue staining, such as SVSPs, as mentioned previously, were observed in high abundance in *B. atrox* and *B. jararaca* venoms due to their recognition by specific antibodies. These results are in agreement with zymographic results, where areas containing high SVSP activity were minimally stained by either blue silver or silver nitrate. The results also demonstrated that BN/SDS-PAGE provided more details of antibody recognition of venom proteins than SDS-PAGE alone (cf. Figure 2 in [62]). Moreover, BN/SDS-PAGE could recognize less abundant venom proteins in more detail than IEF/SDS-PAGE (cf. Figure 8b in [4]).

One limitation of this study is that other techniques—e.g., gel filtration, co-immunoprecipitation, and cross-linking mass spectrometry [30,63,64,65]—were not employed to evaluate the intricate formation of protein complexes in snake venoms. We mostly focused on searching for a simple technique that allowed a more detailed analysis of snake venoms by images. Further modifications of this technique will be required to allow a better evaluation of different types of venoms.

## 3. Conclusions

BN/SDS-PAGE proved to be a fast, low-cost, and effective technique for separating proteins from the venoms of *B. jararaca*, *B. atrox*, and *B. erythromelas* in their native forms, as well as to preserve their proteolytic activities and immunoreactivity. BN-PAGE was shown to be a particularly useful technique for those laboratories with low resources. In particular, the zymography technique described herein for investigating the presence of SVSPs in these snake venoms had a visual benefit in comparison with other exclusively quantitative techniques. Our results provided just a glimpse into the formation of protein–protein interactions in *Bothrops* snake venoms. The era of the identification of new proteins in snake venom has not finished yet, but certainly the focus of future investigations will be how single proteins interact with each other, and the relevance of protein complexes to increase or decrease the pathophysiological effects of envenomation in victims.

## 4. Materials and Methods

### 4.1. Venoms

Pools of lyophilized venoms of adult specimens of *B. atrox*, *B. erythromelas*, and *B. jararaca* were obtained from the Laboratory of Herpetology, Instituto Butantan, São Paulo, SP, Brazil (Sistema Nacional de Gestão do Patrimônio Genético e do Conhecimento Tradicional Associado, SisGen AF375C2). They were maintained and stored at −20 °C and diluted immediately before use.

### 4.2. Reagents

Ponceau S (code P3504), glycerol (code G9012), Nα-Benzoyl-DL-arginine 4-nitroanilide hydrochloride (code B4875), dimethylsulfoxide (DMSO, code 472301), gelatin from porcine skin (code G8150), 1-naphthalamine (code N9005), peroxidase-conjugated anti-horse IgG (code A6917), and sodium nitrite (code 237213) were obtained from Sigma (USA). Bis-acrylamide was purchased from Ludwig Biotec. Tricine (code 22561) was obtained from USB corporation (USA), and agarose (type VII, code V3125) and acrylamide (code V3115) were from Promega. Polyclonal anti-botrocetin antibodies were raised in rabbits [61], and commercial *Bothrops* antivenin was a kind donation of Institute Butantan, São Paulo, SP, Brazil (lot 0611203). AlexaFluor 647-conjugated anti-rabbit IgG (code A21245) was purchased from Invitrogen (USA). Coomassie brilliant blue R-250 (code 161-0400), Coomassie brilliant blue G-250 (code 161-0406), the molecular mass standard (code 61-0376), and 0.22-µm nitrocellulose membranes (162-0112) were obtained from Bio-Rad. HD-Phe-Pip-Arg-pNA (code S-2238) was purchased from Chromogenix (USA). Triton X-100 (code 11869), imidazole (code 4716), and urea (code 57136) were obtained from Merck. Tris-base (code 1027) and glacial acetic acid (code 1019) were obtained from Synth.

### 4.3. BN/SDS-PAGE

The first- (BN-PAGE) and second-dimension (SDS-PAGE) electrophoreses were carried out in a Mighty Small SE250/SE260 vertical electrophoresis system (GE Healthcare/Life Sciences).

First-dimension gel electrophoresis (BN-PAGE): the first dimension was performed as described elsewhere [34]. Briefly, 100 μL of sample buffer (50 mM NaCl, 50 mM imidazole, pH 7.0) and 100 μL of ponceau S solution (0.1% ponceau S, 50% glycerol (*w*/*v*)) were added to venom samples (1 mg). For the electrophoresis run, 10 mL of 12% acrylamide gel solution was prepared by mixing 2.5 mL of AB-3 mix solution (48% acrylamide, 1.5% bis-acrylamide), 3.4 mL of gel buffer 3× (75 mM imidazole, pH 7.0), 4.1 mL of Milli-Q water, 27 μL of 10% ammonium persulfate solution, and 7 μL of TEMED. This solution (8.5 mL) was poured into the space between the glass plate (10 × 10 cm^2^, code SE262P-5, GE Healthcare Life Sciences) and the notched alumina plate (10 × 8 cm^2^, code SE202N-10, GE Healthcare Life Sciences), separated by a pair of 1.5 mm thick spacers. After gel polymerization, the sample application gel solution (3.5% acrylamide, 6 mL) was prepared by mixing 0.44 mL of AB-3 mix, 2 mL of gel buffer 3×, 3.4 mL of Milli-Q water, 50 μL of 10% ammonium persulfate, and 50 μL of TEMED. This solution was poured into the space between the plates, and a 10-well, 1.5 mm-thick, comb (code SE211A-10-1.5, GE Healthcare Life Sciences) was inserted. After gel polymerization, the lower chamber of the electrophoresis system was filled with the anode buffer (25 mM imidazole, pH 7.0), and the upper chamber with B+Coomassie buffer (50 mM tricine, 7.5 mM imidazole and 0.02% Coomassie brilliant blue G-250). The samples (200 µg in 40 µL) were loaded into wells, and then subjected to electrophoresis at 20 mA at room temperature using a PowerPac Power Supply (Bio-Rad, USA). When the blue marker (Coomassie brilliant blue G-250) reached ⅓ of the run (Figure 2a), the cathode B+Coomassie buffer was removed and cathode B/10 buffer (50 mM tricine, 7.5 mM imidazole, and 0.002% Coomassie brilliant blue G-250) was added for better detection of protein bands. After the end of the run, the lanes of each sample were cut with a spatula, so that they were parallel to the direction of the run (Figure 2b,c). The gel strips were stored individually in 15 mL Falcon tubes, and maintained frozen at −80 °C until undertaking the second-dimension electrophoresis.

Second-dimension gel electrophoresis (SDS-PAGE): gel strips taken from the first dimension were incubated in 5 mL of 1% SDS in 0.36 M Tris-HCl buffer (pH 8.8) on a tilting shaker at room temperature for 30 min. Thereafter, strips were rinsed with running buffer (25 mM Tris, 192 mM glycine, 0.1% SDS, pH 8.3), inserted horizontally between the plates, and then the 12% SDS-PAGE running gel was poured (Figure 2d). The strips remained over the running gel. After gel polymerization, 5 μL of the molecular mass standard (Bio-Rad, USA) was deposited on a 2 mm × 2 mm filter paper, and the square was inserted between the plates, and its left side (Figure 2d). Then, agarose solution (0.25% agarose in running buffer), melted in the microwave, was poured between the plates, so that it involved the strip and the filter paper square. When agarose solidification was observed, the electrophoresis run was initiated under constant amperage condition (20 mA), using the running buffer in both the upper and lower chambers [66]. When the dye marker reached the end of the plate, the run was stopped, and the gel was removed. Gels were used in zymography assays for SVSPs or SVMPs, Western blotting, silver nitrate staining [40], or blue silver colloidal Coomassie brilliant blue G-250 staining [38] (Figure 2e). After staining, gels were scanned at 600 dpi resolution in an Imagescanner III (Epson). The R_f_ value was calculated by dividing the migration distance (in milliliters) of the spot of interest, from the top of the resolving gel, by the migration distance (in milliliters) of the dye front.

### 4.4. Zymography

Amidolytic activity: to visualize the catalytic activity of SVSPs, the chromogenic substrate HD-Phe-Pip-Arg-pNA (S-2238) was used, since it showed higher amidolytic activity than BAPNA to *B. jararaca* SVSP [67]. S-2238 is hydrolyzed by SVSPs, and p-nitroaniline (pNA) is released, whose yellow color can be visualized directly in the gel or in nitrocellulose membranes. However, released pNA is more easily visible if reacted with 1-naphthalamine [50,68]. In brief, venom samples (200 µg) were prepared and subjected to BN/SDS-PAGE as described above. After finishing the run, the gels were washed with 2.5% triton X-100 for 15 min, and the procedure was repeated three times to remove SDS from the gel. Nitrocellulose membranes were cut at the size of the gels, and were sequentially wetted in Milli-Q water and then in reactive solution (1 mM S-2238 in 50 mM Tris-HCl, 20 mM CaCl2, pH 8.2). To develop the reaction with S-2238, the membranes were placed on the gel surface for 20 min on a compact heated plate (WTA, code 20435, Campinas, SP, Brazil) at 37 °C. Subsequently, the membranes were washed with 0.1% sodium nitrite for 2 min, 1% urea for 30 s, and incubated in 0.025% 1-naphthalamine solution for approximately 20 min, until the visualization of pink spots occurred [50]. Thereafter, to verify colocalization of pink spots (SVSP activity) and protein spots, the very gels were immersed for approximately 1 h in blue silver staining [38].

Gelatinolytic activity: the proteolysis of denatured collagen copolymerized in SDS-PAGE gels was used [4]. Venom samples (200 µg) were prepared and subjected to BN/SDS-PAGE. The gels were then washed in 2.5% Triton X-100 solution, and thereafter incubated in the developing buffer (50 mM Tris, 5 mM CaCl2, pH 8.8) at room temperature for 15 min, under agitation, and then transferred to an incubator at 37 °C for 18 h. At the end of this step, gels were stained with Coomassie brilliant blue R-250 for 1 h and slowly destained in a destaining solution (43.5% ethanol and 10% glacial acetic acid) for better visualization of bands showing gelatinolytic activity.

### 4.5. Western Blotting

Venom samples (200 µg) submitted to BN/SDS-PAGE were transferred to nitrocellulose membranes in a semi-dry system (Bio-Rad, USA) at 15 V for 2 h. Membranes were blocked with 5% non-fat milk in washing buffer (0.1% Tween 20 in PBS, pH 7.4), incubated at room temperature with polyclonal anti-botrocetin antibodies (1:20,000) for 2 h, and subsequently incubated with AlexaFluor 647-conjugated anti-rabbit IgG (1:2500) for 1 h. The membranes were analyzed in a ChemiDoc MP system (Bio-Rad, USA) and the ImageLab software (version 5.2.1, Bio-Rad, USA) was used to observe reactive spots [61,69]. Thereafter, the same membrane was blocked again with 5% non-fat milk and incubated with *Bothrops* antivenin (1:1000), and subsequently with peroxidase-conjugated anti-horse IgG (1:8000). The reaction was developed with diaminobenzidine (DAB) as previously described [70,71].

### 4.6. Mass Spectrometry (MS) Identification

The four most intensely stained spots from *B. jararaca* venom were excised and in-gel trypsin digestion was performed as described elsewhere [72]. Ten μL volume of sample was loaded on the microUPLC-ESI-qTOF-MS system; microUPLC (microACQUITY ultra pressure liquid chromatography) and XEVO G2 XsQToF mass spectrometer equipped with lockspray ion source). Prior to the injection, the columns were equilibrated with 93% mobile phase A (water with 0.1% formic acid) and 7% mobile phase B (acetonitrile containing 0.1% formic acid). The column temperature was set to 40 °C. First, peptides were trapped on an ACQUITY UPLC Symmetry C18 Trap column (Waters Corp., Milford, MA, USA) 5 μm particle size, 300 μm i.d. 25 mm length, at 15 μL/min flow rate for 4 min. Peptides were separated from the trap column by gradient elution to an analytical column ACQUITY UPLC M-Class HSS T3 column, 1.8 μm particle size, 300 μm i.d. 150 mm length (Waters, Corp., Milford, USA), at 5 μL/min flow rate with a gradient 7% to 40% acetonitrile over 82 min, followed by a 6 min rinse of 85%. The column was re-equilibrated at initial conditions for 24 min. Data independent acquisition mode (MSE) was carried out by operating the instrument at positive ion V mode, applying the MS and MS/MS functions over 0.5 s intervals with 6 V low energy and 15–45 V high energy collision to collect the peptide mass to charge ratio (*m*/*z*) and the product ion information to deduce the amino acid sequence. Capillary voltage and source temperature were set to 3.0 kV and 80 °C, respectively. To correct for the mass drift, the internal mass calibrant leucine enkephalin (556.2771 Da) was infused at every 30 s through the lockspray ion source at 3 μL/min flow rate. Peptide signal data were collected between 100–2000 m/z values.

The resulting fragment spectra were searched using PEAKS Studio 64-bit search engine (Matrix Science, London, UK) against the UniProt/Swiss-Prot *Bothrops jararaca* database (in 5 December 2021), and restricted to fragment tolerance of 0.2 Da. Iodoacetamide derivative of cysteine and oxidation of methionine were specified in PEAKS Studio 64 bit as variable modifications. Peptide identifications were accepted if they exceeded specific database search engine thresholds (*p* < 0.01). Protein identifications were accepted if they contained at least 1 identified peptide.

## Data Availability

Publicly available datasets were analyzed in this study. This data can be found here: https://repositorio.butantan.gov.br/handle/butantan/4468, accessed on 22 September 2022.

## Supplementary Materials

The following supporting information can be downloaded at: https://www.mdpi.com/article/10.3390/toxins14100661/s1, Table S1: Mass spectrometry data from proteins identified in four spots from *Bothros jararaca* snake venom.

## References

[1] Sistema de Informação de Agravos de Notificação (Sinan). www.saude.gov.br/sinanweb.

[2] Campbell J.A., Lamar W.W. (2004). The Venomous Reptiles of the Western Hemisphere.

[3] Santoro M.L., Sousa-e-Silva M.C., Gonçalves L.R., Almeida-Santos S.M., Cardoso D.F., Laporta-Ferreira I.L., Saiki M., Peres C.A., Sano-Martins I.S. (1999). Comparison of the biological activities in venoms from three subspecies of the south american rattlesnake (*crotalus durissus terrificus*, *c. Durissus cascavella* and *c. Durissus collilineatus*). Comp. Biochem. Physiol. C Pharmacol. Toxicol. Endocrinol..

[4] Antunes T.C., Yamashita K.M., Barbaro K.C., Saiki M., Santoro M.L. (2010). Comparative analysis of newborn and adult *bothrops jararaca* snake venoms. Toxicon.

[5] Acunha T., Nardini V., Faccioli L.H. (2021). A lipidomics approach reveals new insights into *crotalus durissus terrificus* and *bothrops moojeni* snake venoms. Arch. Toxicol..

[6] Villar-Briones A., Aird S.D. (2018). Organic and peptidyl constituents of snake venoms: The picture is vastly more complex than we imagined. Toxins.

[7] Eble J.A. (2019). Structurally robust and functionally highly versatile-c-type lectin (-related) proteins in snake venoms. Toxins.

[8] Jia Y., Kowalski P., Lopez I. (2021). Using yeast two-hybrid system and molecular dynamics simulation to detect venom protein-protein interactions. Curr. Res. Toxicol..

[9] Siigur E., Aaspollu A., Trummal K., Tonismagi K., Tammiste I., Kalkkinen N., Siigur J. (2004). Factor x activator from *vipera lebetina* venom is synthesized from different genes. Biochim. Biophys. Acta.

[10] Breithaupt H. (1976). Neurotoxic and myotoxic effects of *crotalus* phospholipase a and its complex with crotapotin. Naunyn Schmiedebergs Arch. Pharmacol..

[11] Tasoulis T., Isbister G.K. (2017). A review and database of snake venom proteomes. Toxins.

[12] Hatakeyama D.M., Tasima L.J., Bravo-Tobar C.A., Serino-Silva C., Tashima A.K., Rodrigues C.F.B., Aguiar W.D.S., Galizio N.D.C., de Lima E.O.V., Kavazoi V.K. (2020). Venom complexity of *bothrops atrox* (common lancehead) siblings. J. Venom. Anim. Toxins Incl. Trop. Dis..

[13] Nuñez V., Cid P., Sanz L., De La Torre P., Angulo Y., Lomonte B., Gutiérrez J.M., Calvete J.J. (2009). Snake venomics and antivenomics of *bothrops atrox* venoms from colombia and the amazon regions of brazil, peru and ecuador suggest the occurrence of geographic variation of venom phenotype by a trend towards paedomorphism. J. Proteom..

[14] Gonçalves-Machado L., Pla D., Sanz L., Jorge R.J., Leitão-De-Araujo M., Alves M.L., Alvares D.J., De Miranda J., Nowatzki J., de Morais-Zani K. (2015). Combined venomics, venom gland transcriptomics, bioactivities, and antivenomics of two *bothrops jararaca* populations from geographic isolated regions within the brazilian atlantic rainforest. J. Proteom..

[15] Nicolau C.A., Carvalho P.C., Junqueira-de-Azevedo I.L., Teixeira-Ferreira A., Junqueira M., Perales J., Neves-Ferreira A.G., Valente R.H. (2017). An in-depth snake venom proteopeptidome characterization: Benchmarking *bothrops jararaca*. J. Proteom..

[16] Jorge R.J., Monteiro H.S., Goncalves-Machado L., Guarnieri M.C., Ximenes R.M., Borges-Nojosa D.M., Luna K.P., Zingali R.B., Correa-Netto C., Gutierrez J.M. (2014). Venomics and antivenomics of *bothrops erythromelas* from five geographic populations within the caatinga ecoregion of northeastern brazil. J. Proteom..

[17] Dawson C.A., Ainsworth S., Albulescu L.O., Casewell N.R., Mackessy S.P. (2021). Snake venom metalloproteinases. Handbook of Venoms and Toxins of Reptiles.

[18] Fox J.W., Serrano S.M. (2005). Structural considerations of the snake venom metalloproteinases, key members of the m12 reprolysin family of metalloproteinases. Toxicon.

[19] Lathan L.O., Staggers S.L. (1996). Ancrod: The use of snake venom in the treatment of patients with heparin-induced thrombocytopenia and thrombosis undergoing coronary artery bypass grafting: Nursing management. Heart Lung.

[20] Lan D., Song S., Liu Y., Jiao B., Meng R. (2021). Use of batroxobin in central and peripheral ischemic vascular diseases: A systematic review. Front. Neurol..

[21] Swenson S.D., Stack S., Markland F.S., Mackessy S.P. (2021). Snake venom phospholipase a_2_ toxins. Handbook of Venoms and Toxins of Reptiles.

[22] Calvete J.J., Sanz L., Perez A., Borges A., Vargas A.M., Lomonte B., Angulo Y., Gutierrez J.M., Chalkidis H.M., Mourao R.H. (2011). Snake population venomics and antivenomics of bothrops atrox: Paedomorphism along its transamazonian dispersal and implications of geographic venom variability on snakebite management. J. Proteom..

[23] Arlinghaus F.T., Eble J.A. (2012). C-type lectin-like proteins from snake venoms. Toxicon.

[24] Takeya H., Nishida S., Miyata T., Kawada S., Saisaka Y., Morita T., Iwanaga S. (1992). Coagulation factor x activating enzyme from russell’s viper venom (rvv-x): A novel metalloproteinase with disintegrin (platelet aggregation inhibitor)-like and c-type lectin-like domains. J. Biol. Chem..

[25] Yamada D., Morita T. (1997). Purification and characterization of a Ca^2+^ -dependent prothrombin activator, multactivase, from the venom of *echis multisquamatus*. J. Biochem..

[26] Solovjov D.A., Platonova T.N., Ugarova T.P. (1996). Purification and characterization of ecamulin—A prothrombin activator from the venom of multi-scaled viper (*echis multisquamatus*). Ukr Biokhim Zh 1978.

[27] Petrovan R.J., Govers-Riemslag J.W., Nowak G., Hemker H.C., Rosing J., Tans G. (1997). Purification and characterization of multisquamase, the prothrombin activator present in *echis multisquamatus* venom. Thromb. Res..

[28] Yamada D., Sekiya F., Morita T. (1996). Isolation and characterization of carinactivase, a novel prothrombin activator in *echis carinatus* venom with a unique catalytic mechanism. J. Biol. Chem..

[29] Cabrera-Orefice A., Potter A., Evers F., Hevler J.F., Guerrero-Castillo S. (2021). Complexome profiling-exploring mitochondrial protein complexes in health and disease. Front. Cell Dev. Biol..

[30] Iacobucci I., Monaco V., Cozzolino F., Monti M. (2021). From classical to new generation approaches: An excursus of -omics methods for investigation of protein-protein interaction networks. J. Proteom..

[31] O’Farrell P.H. (1975). High resolution two-dimensional electrophoresis of proteins. J. Biol. Chem..

[32] Lasserre J.P., Ménard A. (2012). Two-dimensional blue native/SDS gel electrophoresis of multiprotein complexes. Methods Mol. Biol..

[33] Schägger H., Cramer W.A., von Jagow G. (1994). Analysis of molecular masses and oligomeric states of protein complexes by blue native electrophoresis and isolation of membrane protein complexes by two-dimensional native electrophoresis. Anal. Biochem..

[34] Wittig I., Braun H.P., Schagger H. (2006). Blue native page. Nat. Protoc..

[35] Fiala G.J., Schamel W.W., Blumenthal B. (2011). Blue native polyacrylamide gel electrophoresis (bn-page) for analysis of multiprotein complexes from cellular lysates. J. Vis. Exp..

[36] Reisinger V., Eichacker L.A. (2007). How to analyze protein complexes by 2d blue native sds-page. Proteomics.

[37] Schägger H., von Jagow G. (1991). Blue native electrophoresis for isolation of membrane protein complexes in enzymatically active form. Anal. Biochem..

[38] Candiano G., Bruschi M., Musante L., Santucci L., Ghiggeri G.M., Carnemolla B., Orecchia P., Zardi L., Righetti P.G. (2004). Blue silver: A very sensitive colloidal coomassie g-250 staining for proteome analysis. Electrophoresis.

[39] Panfoli I., Calzia D., Santucci L., Ravera S., Bruschi M., Candiano G. (2012). A blue dive: From ’blue fingers’ to ’blue silver’: A comparative overview of staining methods for in-gel proteomics. Expert Rev. Proteom..

[40] Blum H., Beier H., Gross H.J. (1987). Improved silver staining of plant proteins, RNA and DNA in polyacrylamide gels. Electrophoresis.

[41] Gharahdaghi F., Weinberg C.R., Meagher D.A., Imai B.S., Mische S.M. (1999). Mass spectrometric identification of proteins from silver-stained polyacrylamide gel: A method for the removal of silver ions to enhance sensitivity. Electrophoresis.

[42] Wang Y.M., Huang K.F., Tsai I.H. (2014). Snake venom glutaminyl cyclases: Purification, cloning, kinetic study, recombinant expression, and comparison with the human enzyme. Toxicon.

[43] Xu C., Wang Y.N., Wu H. (2021). Glutaminyl cyclase, diseases, and development of glutaminyl cyclase inhibitors. J. Med. Chem..

[44] Kawasaki T., Fujimura Y., Usami Y., Suzuki M., Miura S., Sakurai Y., Makita K., Taniuchi Y., Hirano K., Titani K. (1996). Complete amino acid sequence and identification of the platelet glycoprotein ib-binding site of jararaca gpib-bp, a snake venom protein isolated from *bothrops jararaca*. J. Biol. Chem..

[45] Matsui T., Hori A., Hamako J., Matsushita F., Ozeki Y., Sakurai Y., Hayakawa M., Matsumoto M., Fujimura Y. (2017). Mutant botrocetin-2 inhibits von willebrand factor-induced platelet agglutination. J. Thromb. Haemost..

[46] Sasaki T., Shirai T., Tsukiji N., Otake S., Tamura S., Ichikawa J., Osada M., Satoh K., Ozaki Y., Suzuki-Inoue K. (2018). Functional characterization of recombinant snake venom rhodocytin: Rhodocytin mutant blocks clec-2/podoplanin-dependent platelet aggregation and lung metastasis. J. Thromb. Haemost..

[47] Polgár J., Clemetson J.M., Kehrel B.E., Wiedemann M., Magnenat E.M., Wells T.N.C., Clemetson K.J. (1997). Platelet activation and signal transduction by convulxin, a c-type lectin from *crotalus durissus terrificus* (tropical rattlesnake) venom via the p62/gpvi collagen receptor. J. Biol. Chem..

[48] Murayama N., Hayashi M.A., Ohi H., Ferreira L.A.F., Hermann V.V., Saito H., Fujita Y., Higuchi S., Fernandes B.L., Yamane T. (1997). Cloning and sequence analysis of a *bothrops jararaca* cdna encoding a precursorof seven bradykinin-potentiating peptides and a c-type natriuretic peptide. Proc. Natl. Acad. Sci. USA.

[49] Oguiura N., Correa P.G., Rosmino I.L., de Souza A.O., Pasqualoto K.F.M. (2021). Antimicrobial activity of snake beta-defensins and derived peptides. Toxins.

[50] Lomate P.R., Jadhav B.R., Giri A.P., Hivrale V.K. (2013). Alterations in the *helicoverpa armigera* midgut digestive physiology after ingestion of pigeon pea inducible leucine aminopeptidase. PLoS ONE.

[51] Furtado M.F.D., Maruyama M., Kamiguti A.S., Antonio L.C. (1991). Comparative study of nine *bothrops* snake venoms from adult female snakes and their offspring. Toxicon.

[52] Santoro M.L., do Carmo T., Cunha B.H., Alves A.F., Zelanis A., Serrano S.M., Grego K.F., Sant’Anna S.S., Barbaro K.C., Fernandes W. (2015). Ontogenetic variation in biological activities of venoms from hybrids between *bothrops erythromelas* and *bothrops neuwiedi* snakes. PLoS ONE.

[53] Lotto N.P., de Albuquerque Modesto J.C., Sant’Anna S.S., Grego K.F., Guarnieri M.C., Lira-da-Silva R.M., Santoro M.L., Oguiura N. (2021). The absence of thrombin-like activity in bothrops erythromelas venom is due to the deletion of the snake venom thrombin-like enzyme gene. PLoS ONE.

[54] Paes Leme A.F., Prezoto B.C., Yamashiro E.T., Bertholim L., Tashima A.K., Klitzke C.F., Camargo A.C., Serrano S.M. (2008). *Bothrops* protease a, a unique highly glycosylated serine proteinase, is a potent, specific fibrinogenolytic agent. J. Thromb. Haemost..

[55] Henriques O.B., Lavras A.A.C., Fichman M., Mandelbaum F.R., Henriques S.B. (1958). The proteolytic activity of the venom of *bothrops jararaca*. Biochem. J..

[56] Zaqueo K.D., Kayano A.M., Simoes-Silva R., Moreira-Dill L.S., Fernandes C.F., Fuly A.L., Maltarollo V.G., Honorio K.M., da Silva S.L., Acosta G. (2014). Isolation and biochemical characterization of a new thrombin-like serine protease from bothrops pirajai snake venom. Biomed. Res. Int..

[57] Zaqueo K.D., Kayano A.M., Domingos T.F., Moura L.A., Fuly A.L., da Silva S.L., Acosta G., Oliveira E., Albericio F., Zanchi F.B. (2016). Bbrzsp-32, the first serine protease isolated from *bothrops brazili* venom: Purification and characterization. Comp. Biochem. Physiol. A Mol. Integr. Physiol..

[58] Bhat S.K., Joshi M.B., Ullah A., Masood R., Biligiri S.G., Arni R.K., Satyamoorthy K. (2016). Serine proteinases from *bothrops* snake venom activates pi3k/akt mediated angiogenesis. Toxicon.

[59] Holzer M., Mackessy S.P. (1996). An aqueous endpoint assay of snake venom phospholipase a_2_. Toxicon.

[60] Gutiérrez J.M., Avila C., Rojas E., Cerdas L. (1988). An alternative *in vitro* method for testing the potency of the polyvalent antivenom produced in costa rica. Toxicon.

[61] Thomazini C.M., Sachetto A.T.A., Albuquerque C.Z., Mattaraia V.G.M., de Oliveira A.K., Serrano S.M.T., Lebrun I., Barbaro K.C., Santoro M.L. (2021). Involvement of von willebrand factor and botrocetin in the thrombocytopenia induced by *bothrops jararaca* snake venom. PLoS Negl. Trop. Dis..

[62] Lira M.S., Furtado M.F., Martins L.M., Lopes-Ferreira M., Santoro M.L., Barbaro K.C. (2007). Enzymatic and immunochemical characterization of *bothrops insularis* venom and its neutralization by polyspecific *bothrops* antivenom. Toxicon.

[63] Ó’Fágáin C., Cummins P.M., O’Connor B.F. (2017). Gel-filtration chromatography. Methods Mol. Biol..

[64] Low T.Y., Syafruddin S.E., Mohtar M.A., Vellaichamy A., NS A.R., Pung Y.F., Tan C.S.H. (2021). Recent progress in mass spectrometry-based strategies for elucidating protein-protein interactions. Cell. Mol. Life Sci..

[65] Wang S., Wu R., Lu J., Jiang Y., Huang T., Cai Y.D. (2022). Protein-protein interaction networks as miners of biological discovery. Proteomics.

[66] Laemmli U.K. (1970). Cleavage of structural proteins during the assembly of the head of bacteriophage t4. Nature.

[67] Menezes M.C., Furtado M.F., Travaglia-Cardoso S.R., Camargo A.C., Serrano S.M. (2006). Sex-based individual variation of snake venom proteome among eighteen *bothrops jararaca* siblings. Toxicon.

[68] Božić N., Vujčić Z. (2005). Detection and quantification of leucyl aminopeptidase after native electrophoresis using leucine-p-nitroanilide. Electrophoresis.

[69] Thomazini C.M., Soares R.P.S., da Rocha T.R.F., Sachetto A.T.A., Santoro M.L. (2019). Optimization of von willebrand factor multimer analysis in vertical mini-gel electrophoresis systems: A rapid procedure. Thromb. Res..

[70] Pukac L.A., Carter J.E., Morrison K.S., Karnovsky M.J. (1997). Enhancement of diaminobenzidine colorimetric signal in immunoblotting. Biotechniques.

[71] Santoro M.L., Barbaro K.C., da Rocha T.R.F., Torquato R.J.S., Hirata I.Y., Sano-Martins I.S. (2004). Simultaneous isolation of platelet factor 4 and glycoprotein iib-iiia complex from rabbit platelets, and characterization of specific chicken antibodies to assay them. J. Immunol. Methods.

[72] Shevchenko A., Tomas H., Havlis J., Olsen J.V., Mann M. (2006). In-gel digestion for mass spectrometric characterization of proteins and proteomes. Nat. Protoc..

